# Case Report: TFE3 Positive Xp11.2 Translocation Renal Cell Carcinoma (TRCC) – A Case Study and Review of the Literature

**DOI:** 10.3389/fonc.2021.826325

**Published:** 2022-01-20

**Authors:** Ignacy Miroński, Jan Mateusz Zaucha, Jacek Kowalski, Renata Zaucha

**Affiliations:** ^1^ Medical University of Gdańsk, Gdańsk, Poland; ^2^ Department of Pathomorphology, Medical University of Gdańsk, Gdańsk, Poland; ^3^ Department of Oncology and Radiotherapy, Medical University of Gdańsk, Gdańsk, Poland

**Keywords:** translocation renal cell cancer, TFE3, TRCC, non-clear renal cell carcinoma, microphtalmia-associated transcription factor

## Abstract

Microphthalmia-associated transcription factor renal cell cancer, also known as translocation renal cell cancer, belongs to the group of extremely rare non-clear-cell kidney neoplasms. Their incidence is lower in adulthood than in childhood. The only known risk factor for the development of this tumor is prior chemotherapy. In the operable stage of the disease, the prognosis depends on the status of regional lymph nodes. Interestingly lymph node positivity worsens the prognosis only in the adult patient population. Radical surgical excision is the best therapy in the early stage. The optimal treatment strategy for locally advanced and metastatic disease has not been established, given the lack of evidence in such a rare disease. We present the case of a patient with an aggressive course of this neoplasm treated with temsirolimus, who achieved 10-month control of this neoplasm accompanied by a discussion on other therapeutic possibilities.

## Introduction

Malignant tumors of the kidneys are diagnosed in 3–15 per 100,000 people yearly (1). In addition to the clear cell cancers that account for 80%–85% of all renal cell cancer (RCC) cases, the World Health Organization (WHO) classification of RCC incorporates 12 rare non-clear-cell entities, including acquired cystic disease-associated, clear cell papillary, fumarate hydratase-deficient, succinate dehydrogenase-deficient, tubulocystic, and microphthalmia-associated transcription factor (MiT) family translocation RCC (TRCC) (2, 3). TRCC was established as a separate entity in 2004 after the discovery of a distinct molecular signature characterized by mutations in the transcription factor 3 or B (*TFE3* or *TFEB*) genes (3–5). Several recognized types of *TFE3* rearrangements are responsible for a wide range of morphologies in TRCC, which frequently make TRCC similar to more common RCC subtypes or neoplasms such as perivascular epithelioid cell tumor or epithelioid angiomyolipoma of the kidney (6). Although the histopathologic and genetic features of these uncommon neoplasms have been described, data on clinical behavior, optimal systemic therapy, and prognosis are limited. In children, TRCC accounts for about 20%–25% of all RCC cases. Although they are rare in adulthood, some cases have been misdiagnosed (5–9). Kuthi et al. found 28 cases of TRCC among 2,804 malignant kidney tumors (10). The incidence is higher in women than in men at a ratio of 1.4–2:1, and the peak age is 20 to 30 years (7, 8). These tumors are usually found incidentally in asymptomatic patients. While radical surgical excision is the best therapy in the early stage, the optimal treatment strategy for locally advanced and metastatic disease has not been established. Therefore, the only way to choose the optimal treatment is based on data collected from small studies or even case reports. In the advent of improved personalized therapies for patients with clear cell renal cancer, detailed case descriptions on the type and effect of various therapies are of utmost importance.

To broaden the knowledge about this infrequent neoplasm, we report a case of an adult patient with advanced TRCC that rapidly progressed after surgical removal of the tumor. The informed consent for publishing this case was obtained from the patient.

## Case Report

In May 2019, after a several-month history of nausea and vomiting and self-resolving episodes of hematuria, two weeks of severe left flank pain motivated a 68-year-old woman to visit her primary physician who ordered a computed tomography (CT) evaluation. CT showed a large (13 × 17 × 19 cm) mass in the left kidney that caused hydronephrosis of the left renal pelvis. The perinephric tissue left renal vein and left suprarenal gland were infiltrated. Several enlarged retroperitoneal lymph nodes (LNs) and a small lesion in the first lumbar vertebra (L1) were not typical but suspected of metastasis. In June 2019 the patient was admitted to the hospital for further evaluations and treatment. The chest X-ray image was normal. Laboratory blood tests showed microcytic anemia with 7.7 mg/dL hemoglobin (Hb), 24% hematocrit (Hct), 78fL Mean Corpuscular Volume (MCV), 1.58 mg/dL serum creatinine, and an impaired creatinine clearance (CrCl) rate of 33 mL/min. All other laboratory results were within normal ranges. The patients’ performance status was reasonably good (PS1). Comorbidities included well-controlled hypertension and hypercholesterolemia. The medical history included endometrial polypectomy and left submandibular gland removal for a benign adenoma, and about seven pack-years of smoking. There was no history of alcohol use, drug abuse or incidence of cancer in the family.

**Figure 1:**
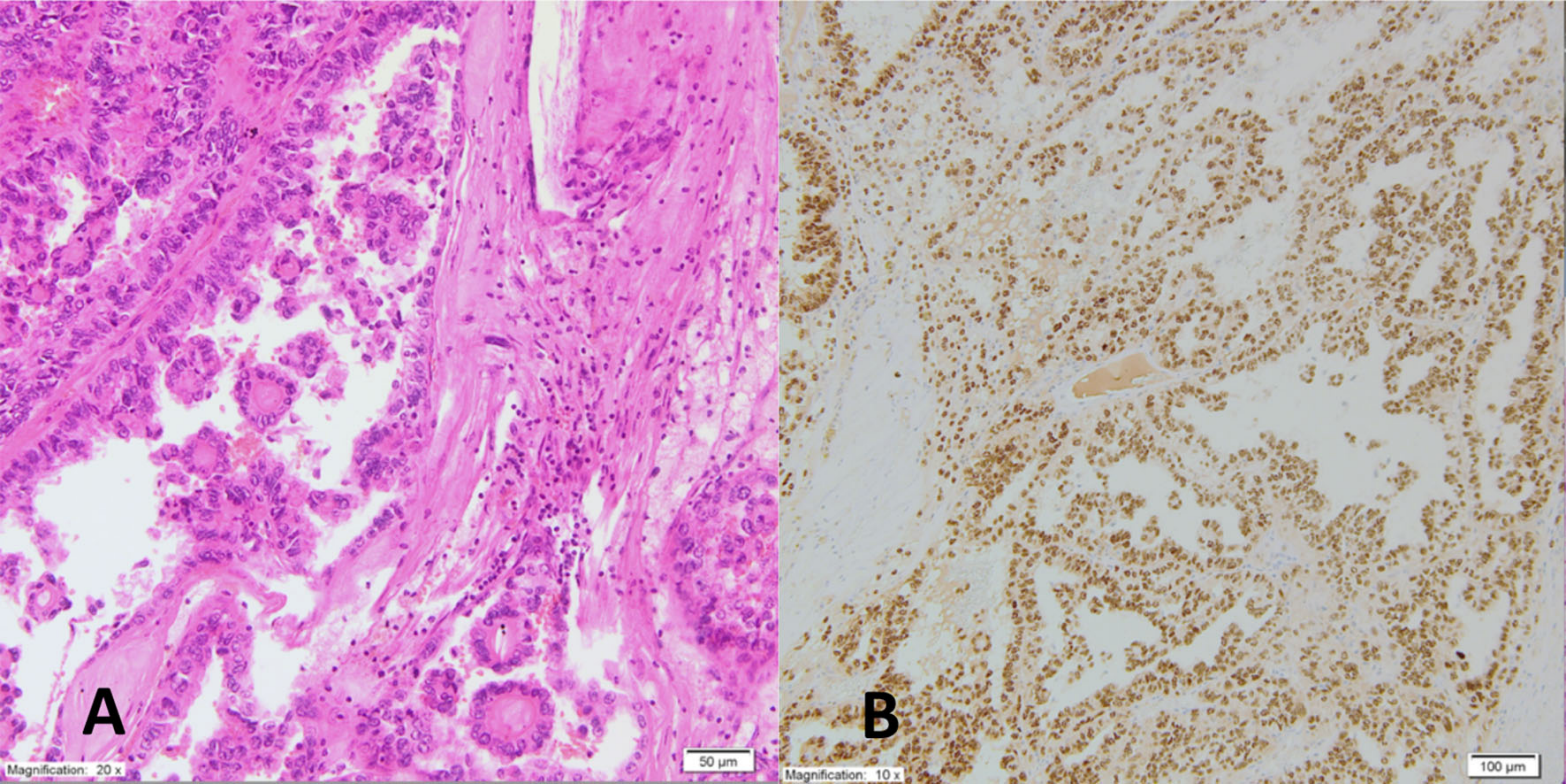
Pathomorphologic picture of the tumor shows **(A)**. on HE examination epithelioid clear cancer cells with nuclear atypia forming papillary and tubular structures; **(B)** on IHC evaluation positive TFE3+ expression.

The patient was qualified for surgical treatment to obtain a pathological diagnosis and enable systemic therapy, which is available only for patients after at least partial nephrectomy in our country. In June 2019, a radical operation removed the left kidney with the tumor, the left adrenal gland, and, to increase the chance for obtaining clear surgical margins, with the spleen. The tumor, necrotic in more than 50%, vastly infiltrated the perinephric fat tissue and veins. A single metastatic nodule was found in the left adrenal gland. The renal pelvis, artery, and vein and all surgical margins were free of cancer. No LNs were observed in the specimen. The tumor was composed of epithelioid clear cancer cells that formed papillary and tubular structures. There was profound nuclear atypia. Immuno-histochemical (IHC) evaluation showed that the tumor was CD10+, vimentin+, TFE3+, and CK7- (**Figure 1**), which led to the final diagnosis of TRCC further confirmed as the Xp11 translocation subtype by fluorescence *in situ* hybridization (FISH) at the pathological stage T3aNxM1 (according to the TNM classification of malignant tumors, 8th edition).

**Figure 2:**
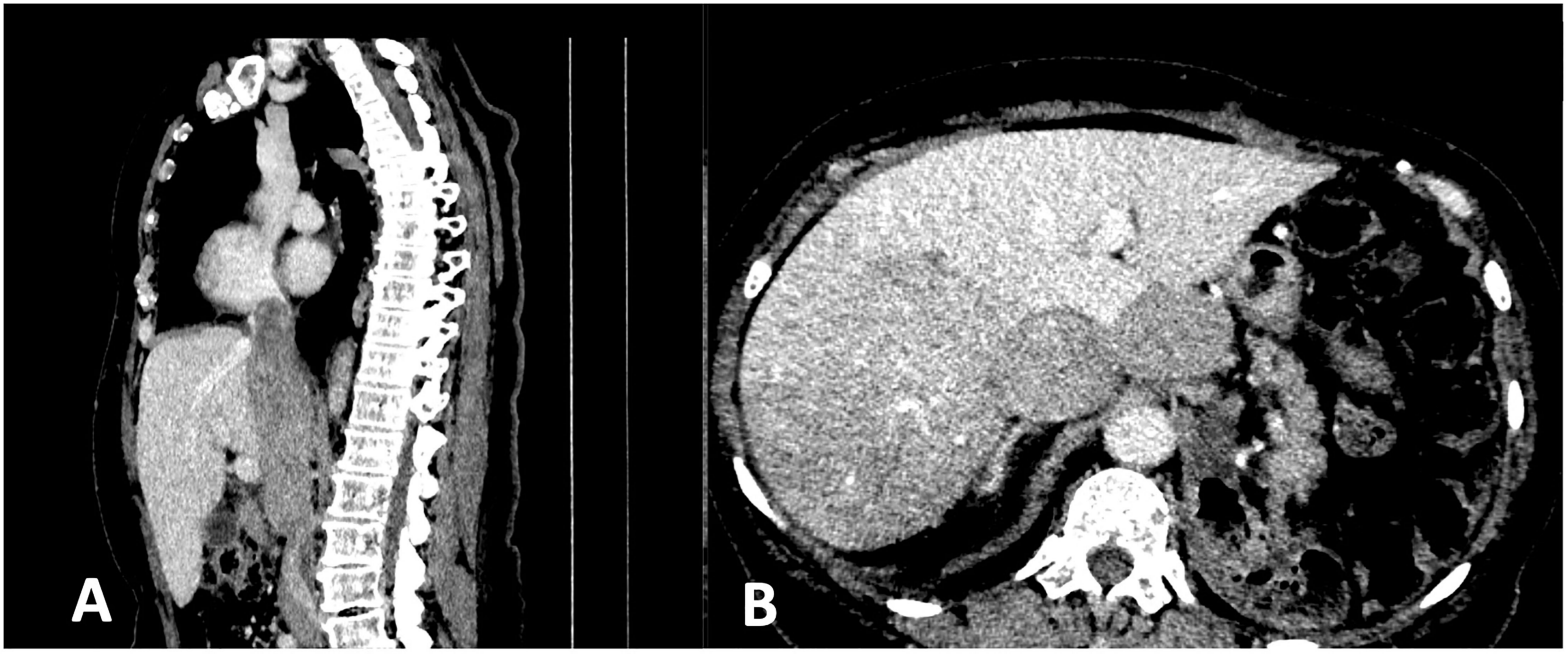
CT scan shows **(A)** the neoplastic thrombus almost completely obstructing inferior vena cava from L2/L3 to the right atrium; **(B)** perfusion changes in the liver.

The early postoperative period was complicated by acute gastrointestinal bleeding; however, neither endoscopy nor CT revealed the bleeding site. The patient recovered after transfusion (with two packs of red blood cells, Hb level increased from 6.7 to 9.1 mg/dL). Echocardiography showed a distended inferior vena cava almost entirely obstructed by a large thrombus. An electrocardiogram, Doppler ultrasonography of the leg veins and the left ventricular ejection fraction (57%) were normal. Beta-blockers (25 mg QD metoprolol) and daily subcutaneous injections of low molecular weight heparin (enoxaparin at a dose adjusted for the decreased CrCl, 40 mg QD) were administered. During the first postoperative visit, the patient reported persistent fatigue and exacerbation of lumbago-like back pain uncontrolled by non-steroidal anti-inflammatory medications. A postoperative CT scan revealed an extensive inoperable, disseminated recurrence with multiple metastases in both lungs, peritoneum, intraabdominal and retroperitoneal LNs and the spine (Th12, L1, and L2). Neoplastic thrombus almost completely obstructed inferior vena cava from L2/L3 to the right atrium with by-pass circulation below and above the diaphragm and perfusion changes in the liver (**Figure 2**). Palliative radiotherapy (RTH) was administered to the affected spine (Th12-L2, 20 Gy in 5 fractions) with symptomatic improvement. Two weeks later, the patient started systemic therapy. Due to the lack of treatment guidelines for rare RCC subtypes, intravenous temsirolimus (25 mg weekly) was initiated as the only available in the country therapy for the non-clear-cell RCC. Metoprolol was switched to carvedilol and amlodipine, and furosemide was added to control exacerbated hypertension. However, mucositis, nausea, diarrhea, appetite decrease, and hypertriglyceridemia led to one-level dose de-escalation (to 20 mg weekly). Anemia was managed with packed RBC transfusion (Hb level increased from 5.7 to 9.0 mg/dL). Thirty-four cycles of therapy were administered and were associated with stabilising the disease (per RECIST 1.1 criteria on CT) without significant complications. Ten months after the start of treatment, a subsequent CT scan showed progression of preexisting lesions and new metastases. A tumor mass in the spine (Th3) caused spinal compression that required urgent palliative radiotherapy (20 Gy in 4 fractions), accompanied by symptomatic improvement. Thus, temsirolimus was withdrawn.

Standard second-line therapy for non-clear cell RCC has not been established. Therefore, in our country, such therapy is regarded as experimental and is not reimbursed. The patient was offered the best supportive care and died in May 2021.

## Discussion

The MiT family of TRCC is one of the five main subtypes of miscellaneous non-clear cell RCC originating from the proximal convoluted tubule of the kidneys. It is characterized by different histological features and clinical behavior related to a unique genetic alteration. The rarity of non-clear-cell RCC precludes the completion of prospective clinical trials, all of which have been prematurely closed due to slow accrual. We report a case of aggressive TRCC in a 68-year-old woman who achieved 10-month progression-free survival (PFS) on temsirolimus. Unlike 15% of the cases with the Xp11.2 translocation subtype of TRCC, our patient had no history of prior chemotherapy (11). The patient deferred evaluation for intermittent hematuria that lasted for several months. The diagnosis was triggered by lumbago-like symptoms caused by the enormous size of the primary tumor, which microscopically infiltrated the spine. Not surprisingly, the disease was discovered at an advanced stage. The histological proof of TRCC was established based on positive IHC expression of TFE3 and CD10 and negative expression of CK7 (11, 12). TFE3 is ubiquitously expressed in normal human cells, although its levels are undetectable. The translocation of the *TFE3* gene becomes detectable after it causes overexpression of the TFE3 protein (12). Vimentin expression is usually negative in TRCC; thus, its positivity in our patient required additional molecular analysis using next generation sequencing to rule out the risk of a false diagnosis.

The histomorphology of adult-onset TRCC has been described in over 400 molecularly proven cases. The involvement of chromosome X (Xp11.2) suggests female preponderance; however, insufficient data confirms this hypothesis. Diverse genetic aberrations such as translocations or inversions of various genes (most frequently PRCC, ASPL, and SFPQ) lead to fusion genes responsible for the histological and clinical differences (6, 9–12). None of the numerous chimeric TFE3 fusion proteins has been useful as therapeutic targets. In addition, their types do not explain the differences in incidence rates and prognoses between children and adults. In a large study by Ma et al. that included 8,001 adults and 82 children, the proportion of pediatric and adult Xp11.2 TRCCs was 20.7% (17 of 82 children) and 0.9% (73 of 8001 adults), respectively. The five-year overall survival (OS) and PFS rates in children were significantly better than those in adults (OS: 75.0% vs. 40.3%, p <0.01; PFS: 64.8% vs. 0%, p=0.04) (4, 8, 9). Interestingly Malouf et al. have found a subgroup with 17q gain responsible for activating the cytotoxic T lymphocyte-associated protein 4 (CTLA4) pathway (13). It is also essential to emphasize the frequent expression of programmed death ligand 1 (PD-L1) in tumor-infiltrating immune cells present in 90% of TRCC cases (14).

LN positivity (LN+) is more common in children (58.8%) than in adults (28.8%, P=0.02) (4). Interestingly, LN+ is associated with significantly shorter PFS (Hazard ratio [HR]=0.10, 95% confidence interval [CI] 0.02–0.51, p=0.01) and OS (HR=0.11, 95% CI 0.01–0.98, p=0.04) but had no impact on prognosis in children (4). In our case, the postoperative specimen did not contain any LNs. The first post-resection CT scan revealed multiple LN metastases.

We noted acute clotting disorders with life-threatening mucosal gastric hemorrhage in our patient apart from diagnostic and staging difficulties.

However, the most critical problem was establishing an optimal treatment plan, given the lack of effective methods and the presence of enormous, possibly neoplastic thrombus in the VCI.

There are various therapeutic options for advanced or metastatic clear cell RCC (clear RCC), which include monotherapy or combinations of immune, antiangiogenic, and signal transduction-blocking agents. Treatment choice depends on the patient’s general status, parenchymal organ function, intensity and evolution of symptoms, and comorbidities. Adjuvant treatment with pembrolizumab has been approved in the USA for high-risk clear RCC, including completely resected metastatic disease because of the significant positive impact on disease-free survival (15). Despite the poor prognosis of TRCC and high expression of PDL-1 in most of cases, adjuvant systemic immunotherapy is not recommended. Moreover, the evidence for the efficacy of available systemic therapies for non-clear RCC is not very strong. The results of the ARCC trial, one of the first randomized controlled phase III trials that included non-clear RCC patients, compared temsirolimus and interferon treatments and favored temsirolimus. However, this agent should be considered with caution because of the low number of non-clear RCC patients (73 of 626 patients with poor-risk non-clear RCC or unconfirmed histology) and suboptimal efficacy of the interferon treatment as a comparator (16).

Other studies, which included patients with non-clear cell histologies, have demonstrated that first-line anti-VEGF therapy with sunitinib offers better outcomes than the mammalian target of the rapamycin (mTOR) inhibitor everolimus (**Table 1**) (17–24). Two studies, ESPN (19) and ASPEN (20), were explicitly conducted in non-clear RCC and confirmed improved PFS and OS with frontline sunitinib therapy. Sequential therapies with sunitinib followed by everolimus or vice versa were evaluated in a trial called RECORD-3 (25). The outcomes favored initial therapy with sunitinib. The three studies showed a median PFS of 6.1, 7.2, and 8.3 months, respectively, much shorter than the median PFS of approximately 11–12 months observed with sunitinib treatment for clear RCC. In a phase II trial of pazopanib the disease control rate for treating non-clear RCC was 81%, and partial responses were observed in 10 of 37 (27%) patients (26). The median PFS and OS were 15.9 and 17.2 months, respectively. The International Metastatic RCC Database Consortium evaluated the outcomes of anti-VEGF therapy in 337 non-clear RCC cases (27). The median OS was 15.7 months in non-clear RCC and 20.2 months in clear RCC. The intermediate- and poor-risk patients predominantly contributed to the differences in OS outcomes between the groups. Outcomes were similar in the favorable group. Single-agent studies of everolimus, c-Met inhibitors, and chemotherapy have also demonstrated modest efficacy. Non-clear RCC, similar to other neoplasms, is driven by various factors such as the MET pathway in papillary kidney cancer or alterations in mitochondrial DNA in chromophobe cancer. However, no essential biological pathway was found in translocation-associated subtypes until 2018. The first study by Argani et al. (28) showed upregulation of the mTOR and HIF-1α pathways. Damayanti et al. (29) repeated these results eight years later. They showed that the TFE3/IRS-1/PI3K/AKT/mTOR pathway is altered in TRCC, suggesting a potential benefit from inhibiting this axis with a dual PI3K/mTOR inhibitor. However, the findings of these studies are limited by the low number of TRCC cases diagnosed annually around the world.

**Table 1:** Treatment results in clinical trials including patients with TRCC.

Study (type)	Agent	Prior treatmentsN	Patients with TRCC/totalN	Ageyears(range)	OSmedian (95%CI)(months)	PFSmedian (95%CI)(months)	Response RECIST 1.1N	ORR%	DCR%
Lee et al. (Retrospective) (17)	Sunitinib	0-1	1/31	53 (18-76)	NA	NA	SD:1	0	100
Tannir et al. (Prospective) (18)	Sunitinib	0-≤2	1/57	57 (22-85)	NA	1.0	PD:1	0	0
Tannir et al. (RCT) (19)	Sunitinib Versus Everolimus	0-1	3/33 4/35	60 (28–76) 58 (23-73)	16.2 (8.8–NA) 8.1 (5.5–23)	6.1 (6.0–8.8) 3.0 (1.3–NA)	NA NA	NA NA	NA NA
Armstrong et al. (RCT) (20)	Sunitinib Versus Everolimus	0	6/51 2/64	59 (24–100) 64 (29–90)	13.2 (9.7-37.9) 31.5 (14.8-NR)	5.5 (3.2-19.7) 11.4 (5.7-19.4)	NA NA	NA NA	NA NA
Koshkin et al. (Retrospective) (21)	Nivolumab	0-3	1/41	58 (33–82)	NR	NA (3.5)	PD:1	0	0
McKay et al. (Retrospective) (22)	PD-1/PD-L1	0-3	3/43	57 (24-75)	12.9 (7.4-NR)	NA	PR:1 SD:1 PD:1	33	66
Campbell et al. (Retrospective) (23)	Cabozantinib	≥0	2/30	58.4 (25-81)	25.4 (15.3-35.4)	8.6 (6.1 - 14.7)	PD:1 SD:1	0	50
Boileve A et al. (Retrospective) (24)	ICIs (anti-CTLA4, anti-PD-1, anti-PDL-1, (monotherapy or combined)	1	24/24	34 (3-79)	24 (4-84.6)	2.5 (1-40)	PR 4 SD 3	29	NA

RCT, randomized clinical trial; N, number; OS, overall survival; PFS, progression free survival; ORR, objective response rate; DCR, disease control rate; NA, not assessed; NR, not reached; PR, partial remission; SD, stable disease; PD, disease progression.

The most current non-clear cell RCC treatment results were released as conference presentations. Lee C-H et al. (30) used nivolumab with cabozantinib in patients who were treatment-naïve or previously treated with either a VEGF-R TKI or mTOR inhibitor for papillary RCC, MiT family TRCC, or unclassified RCC. Patients showed an overall response rate (ORR) of 48% (95% CI 31.5%–63.9%), a median PFS of 12.5 (95% CI 6.3–16.4) months, and an OS of 28 (95% CI 16.3–not established NE) months with an acceptable toxicity profile. The second cohort (for the chromophobe subtype) was closed early due to a lack of efficacy (ORR 0%), as previously suggested by the KEYNOTE 427 trial (31).

It is impossible to conclude ICI efficacy from other clinical trials as TRCC was not explicitly addressed; however, patients with TRCC may have been included among the unclassified cases.

Among other targets, the highest level of evidence has been shown for the efficacy of MET inhibitors in non-clear RCC. The results of the SWOG 1500 study (32) comparing sunitinib against three MET inhibitors (cabozantinib, crizotinib, and savolitinib) favored cabozantinib, which was suggested as the preferred frontline treatment. However, the study exclusively involved patients with papillary RCC, so the results cannot be extrapolated to other subtypes (32). Treatment with cabozantinib (40 mg QD orally) in combination with atezolizumab (1200 mg iv Q3W) assessed in the COSMIC-021 study (33) was associated with an ORR of 27% (95% CI 19%–36%) across all non-clear RCC histologies (of 112 patients, 66 (59%) had papillary RCC, 17 (15%) had TRCC, 15 (13%) were unclassified, ten (9%) had the chromophobe subtype, and four (4%) had collecting duct histology). Among the 54 patients with available next-generation sequencing data, the most frequently altered somatic genes were CDKN2A (22%) and MET (20%), and responses were seen irrespective of mutational status. At a median follow-up of 11 months, the median time to treatment failure, median PFS, and median OS were 6.7 (95% CI 5.5–8.6) months, 7.0 (95% CI 5.7–9.0) months, and 12.0 (95% CI 9.2–17.0) months, respectively, with relatively good tolerance and no treatment-related deaths. Unfortunately, TRCC cases were not included in this study. Thus, cabozantinib seems to have the most significant impact on patient outcomes in MET-driven disease.

The interaction between RCC and the immune system was noticed many years ago. Studies on the “immune escape” mechanisms have led to investigating PD- 1/PD-L1- and CTLA-4-targeted immune checkpoint inhibitors (ICIs) to treat RCC. While ICIs have been studied in clear RCC since 2015, their effects in non-clear cell subtypes have only recently been highlighted in the 2021 phase II KEYNOTE 427 trial (31). With over 100 non-clear RCC patients, the response rate to anti-PD-1 treatment was 25%. This was similar to the 29% disease control rate and 16,7% objective responses in the international, multicenter retrospective study of 24 patients with metastatic MITF family TRCC treated with ICIs (24). Currently, the activity of nivolumab with or without ipilimumab is being studied in non-clear cell histotypes (UNISoN, NCT03177239, SUNIFORECAST, and NCT03075423). Several other immunotherapeutic strategies are being investigated and include combinations of anti-PD-1 antibodies with modified NK cells (NCT04551885), D-CIK cells (a heterogeneous subset of ex-vivo expanded T lymphocytes; NCT03987698), agonists or inhibitors of cytokines (polyethylene glycosylated IL-2; NKTR-214), dendritic cell-based immunotherapy, anti-LAG-3, anti-Tim-3, anti-CD27 mAb, metabolism-related molecules, individualized cancer vaccines, and several other agents.

Promising data from single-arm phase 2 studies warrant randomized evidence. Therefore, next-generation studies will probably be based on combinations of anti-Met agents with immunotherapy for an additive, if not synergistic effect.

Our case is unique because of a long time of stabilization of the disease achieved by temsirolimus - the only medication available in our country for non-clear RCC. Based on published reports, sunitinib is superior to mTOR inhibitors like temsirolimus or everolimus. However, there were significant medical contraindications for anti-VEGF therapies, including sunitinib. Keeping all those factors in mind, everolimus/temsirolimus or ICIs, if available, were the best. These treatment options are not correlated with such frequent adverse events as clotting disorders.

We provided the only treatment that was available for non-clear cell kidney cancer. We regret that the patient did not have a chance to benefit from other promising treatment options like ICIs.

## Conclusion

Continued international collaborations and ongoing prospective studies focusing on TRCC cases with specific molecular alterations are warranted to improve clinical outcomes for this rare disease, with few evidence-based treatment options.

Because prospective studies including rare RCC subtypes, including TRCC, will take years to produce results, retrospective studies or case reports can inform treatment decisions.

## Data Availability Statement

The original contributions presented in the study are included in the article/**Supplementary Material**. Further inquiries can be directed to the corresponding author.

## Ethics Statement

Written informed consent was obtained from the individual(s) for the publication of any potentially identifiable images or data included in this article.

## Author Contributions

Students IM and JZ: collected data, searched the literature and wrote the manuscript. JK: prepared histopathological examination and illustrations. RZ: conceived the manuscript, consulted the treatment plan, reviewed the topic presentation, structure of the manuscript, illustrations and photographs. All authors contributed to the article and approved the submitted version.

## Conflict of Interest

The authors declare that the research was conducted in the absence of any commercial or financial relationships that could be construed as a potential conflict of interest.

## Publisher’s Note

All claims expressed in this article are solely those of the authors and do not necessarily represent those of their affiliated organizations, or those of the publisher, the editors and the reviewers. Any product that may be evaluated in this article, or claim that may be made by its manufacturer, is not guaranteed or endorsed by the publisher.
