# Supplementary material for: Case Report: TFE3 Positive Xp11.2 Translocation Renal Cell Carcinoma (TRCC) – A Case Study and Review of the Literature
Source: Front Oncol. 2022 Jan 20;11:826325. doi: 10.3389/fonc.2021.826325 (PMC8812275; doi:10.3389/fonc.2021.826325)
Supplement: Supplementary file 1 [file DataSheet_1.docx]

**PROGRESSION**
